# Seed Dormancy Breaking and Germination in *Bituminaria basaltica* and *B. bituminosa* (Fabaceae)

**DOI:** 10.3390/plants9091110

**Published:** 2020-08-27

**Authors:** Francesca Carruggio, Andrea Onofri, Carmen Impelluso, Gianpietro Giusso del Galdo, Giovanni Scopece, Antonia Cristaudo

**Affiliations:** 1 Department of Biological, Geological and Environmental Sciences, Germplasm Bank (BGS-CT), University of Catania, 95128 Catania, Italy; francesca.carruggio@unict.it (F.C.); carmen.imp@libero.it (C.I.); 2 Department of Agricultural, Food and Environmental Sciences, University of Perugia, 06121 Perugia, Italy; andrea.onofri@unipg.it; 3 Department of Biological, Geological and Environmental Sciences, Hortus Botanicus Catinensis, University of Catania, 95125 Catania, Italy; g.giusso@unict.it; 4 Department of Biology, University of Naples Federico II, Complesso Universitario MSA, 80126 Naples, Italy; giovanni.scopece@unina.it

**Keywords:** physical dormancy, seed coat structure, scarification techniques, pod maturity stages

## Abstract

Most legumes are well-known for the physical dormancy of their seeds; hence, the implementation of appropriate scarification techniques is essential for introducing new legume crops within agricultural systems. This study investigated morpho-anatomical traits and dormancy-breaking requirements in two taxa of the genus *Bituminaria:* the widespread *B. bituminosa* and the point endemic *B. basaltica*. As the species under investigation show monospermic indehiscent legumes, pods were used in this research. We performed pod trait measurements, light microscopy observations on the seed coat anatomical structure, and germination tests after mechanical, thermal, and chemical scarification treatments for seed dormancy breaking. Moreover, germination performance at different pod maturity stages and storage times was tested. Differences in morpho-anatomical traits were found, with *B. basaltica* having a thicker palisade cell layer and *B. bituminosa* showing larger pods. All of the scarification treatments proved to be able to break physical dormancy, with mechanical and chemical scarification being the most effective methods in both species. Nevertheless, dormancy-breaking treatments performed better in *B. bituminosa*. Seeds at early pod maturity stages showed higher germination capacity in both species. Overall, this research provided background knowledge on seed collection time, storage strategy, and effective pre-sowing treatment, which might contribute to enhance propagation and use of *Bituminaria* species for multiple purposes. Under this perspective, the future characterization of additional *Bituminaria* genetic resources from other Mediterranean populations will have remarkable importance.

## 1. Introduction

In the Mediterranean region, natural pasture areas possess a considerable component of native legume species [1]. This is a very remarkable characteristic because these plants are both a source of highly nutritive natural forage, and an effective nitrogen fixation agent into the soil [2]. Therefore, an increasing interest is being given to the identification of new wild genetic resources within this plant family to be used as forage and for soil enrichment in marginal areas [3]. Drought tolerant legume species appear to be particularly promising for those purposes, as their use can extend grazing potential in marginal areas, and may thus represent an element of resilience to climate changes, which are expected to increase the extension of arid areas [4].

The introduction of new legume genetic resources requires the characterization of their morpho-agronomic traits, which should be preceded by the assessment of their seed germination requirements, as the main prerequisite for planning long-lasting pasture amelioration strategies [5]. Indeed, it is well known that many legume species exhibit physical dormancy (PY) and only germinate after exposure to specific environmental conditions [6].

PY is an important adaptive trait that allows to delay germination and seedling emergence until favorable environmental conditions occur [7]. This mechanism is particularly common in habitats with high seasonal or inter-annual climate variability and contributes to the maintenance of a persistent soil seed bank. However, similarly to other classes of dormancy *sensu* Baskin and Baskin [8], PY may be an undesirable trait for agricultural purposes.

For this reason, seed dormancy and germination patterns in legume species have been long since investigated, mainly in wild annual taxa (e.g., *Lotus*, *Trifolium*, *Medicago,* and *Scorpiurus*) [9,10,11,12]. Annual legumes have an elevated capacity of self-reseeding, but also low levels of germinability [13], a trait that was found to be linked to a strong PY mechanism, preventing seeds from achieving rapid germination, optimal emergence and uniform seedling establishment [6,8,14]. From an anatomical perspective, PY is due to the oxygen- and water-impermeability of the seed coat, which has phenolics- and suberin-impregnated layers of palisade cells (outer macrosclereids) [6,7,15]. Seed coat impermeability is acquired during the later stages of maturation, when seed moisture content falls below a specific threshold level (14% approximately) [16,17,18].

Many scarification treatments have been explored to break PY in legumes, but the use of acid substances appears to be the most effective method [12,19,20]. Indeed, such treatment allows a significant increase of germination in many Fabaceae [7,21,22].

Most of past studies were mainly focused on annual legumes that, however, have a major drawback, as they disappear during the long and dry Mediterranean season due to their short life cycle [23]. More recently, therefore, an increasing interest has been arising for wild perennial legumes, searching for summer-active species that have the additional important characteristic of maintaining green biomass during the driest years [24]. These species could hence be a promising resource to improve sustainability of grazing management targets, providing additional crop rotation options in view of climate changes [3,4,25]. Likely, the most interesting group is the genus *Bituminaria*, which is a fairly complex and poorly investigated taxonomical group spanning from the Mediterranean Basin to the Macaronesian Islands [26,27]. Few *Bituminaria* species have received some attention as drought resistant plants [28,29], well-adapted to different soil types [30] and even to heavy metal contaminated sites [31,32]. A marked chemical and morphological variability between populations has been highlighted within the genus that could be an important genetic resource for multiple purposes (i.e., forage production in Mediterranean environments, pharmaceutical industry, phytoremediation, biofertilization, and rehabilitation of degraded areas) [33,34,35,36,37,38].

Within the *Bituminaria* genus, studies on seed germination have been conducted in *B. bituminosa* from the Canary Islands [39,40,41], continental Spain [39,42], and Morocco [43]. These studies confirmed that PY also characterizes the *Bituminaria* genus, hindering germination [39,42,44]. Besides, data on the occurrence of physiological dormancy is also available [40]. This body of data clearly shows that the germination behavior of *Bituminaria* species can be rather variable, also depending on geographic provenances. However, information is still scattered and incomplete, making it crucial to characterize seed germinability of further potentially interesting taxa, also enhancing genetic resources of additional Mediterranean populations.

Sicily represents a ‘hotspot’ for *Bituminaria* biodiversity including the widespread *B. bituminosa* and several local segregates often located in small islands [45]. This biodiversity richness could represent an important source for promising *Bituminaria* genotypes and should be therefore investigated. In this context, the present study was aimed at evaluating the germination response of two *Bituminaria* species from Sicily, i.e., *B. bituminosa* and *B. basaltica*, the latter being a point endemic species to the small island of Filicudi (Aeolian Archipelago). Both the species show monospermic indehiscent legumes, which act as the dispersion unit. Although pods were used in our research, the word seed is also employed throughout the text when referring to germination and dormancy, since the last two terms are intrinsically linked to seed behavior. We collected pods in the wild from natural populations and performed germination tests in the laboratory on parental material, to address the following specific questions: (1) do *B. basaltica* and *B. bituminosa* seeds exhibit PY?; (2) when is dormancy established during seed development?; (3) do mechanical, thermal, and chemical scarification treatments allow to overcome seed dormancy in the two investigated species?; (4) are there differences in the response of these species to dormancy-breaking treatments and, if any, are these differences linked to morpho-anatomical seed traits?

## 2. Results

### 2.1. Morphometric Analysis, Seed Coat Structure, and Water Imbibition Test

**Figure 1:**
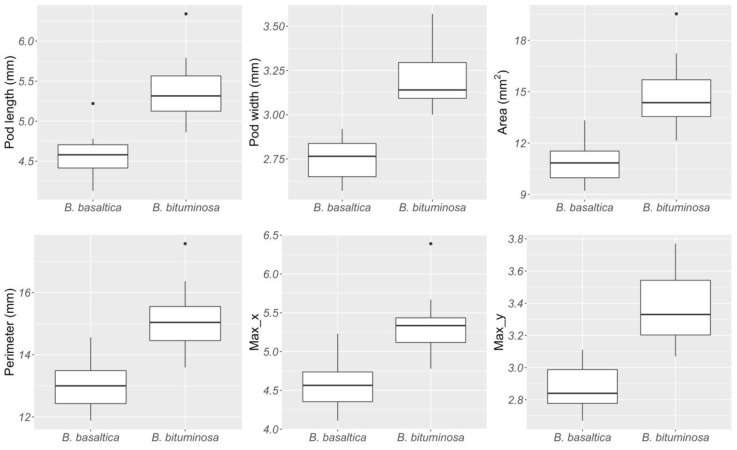
Box plot showing the mean average values of different pod morphometric traits in *B. basaltica* and *B. bituminosa*. Max X = major axis of the ellipse; Max Y = minor axis of the ellipse.

The two species under investigation produce monospermic pods of variable mass and size. Brown dehydrated (BD) pod mass was significantly lower in *B. basaltica* than in *B. bituminosa* (16.36 and 24.89 mg, respectively; t = −23.246, Satterthwaite Degrees of Freedom = 4.5, *p* < 0.0001). *B. basaltica* pods were generally smaller than those of *B. bituminosa*. All the six morphometric descriptors were significantly different between the two species (Length: t = −4.90, DF = 16.1, *p* = 0.0002; Width: t = −6.820, DF = 16.5, *p* < 0.0001; Area: t = −4.968, DF = 14.3, *p* = 0.0002; Perimeter: t = −4.827, DF = 16.2, *p* = 0.0002; Max X = Major axis of the ellipse: t = −4.587, DF = 16.3, *p* = 0.0003; Max Y = Minor axis of the ellipse: t = −5.396, DF = 14.8, *p* = < 0.0001; Figure 1).

**Figure 2:**
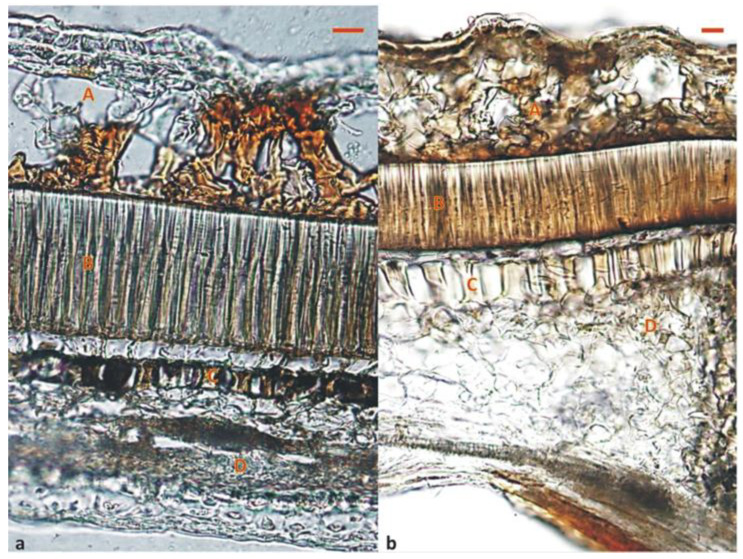
Longitudinal sections of the pods of (**a**) *B. basaltica* and (**b**) *B. bituminosa*. A = cellular structure of the pod wall (pericarp); B = palisade cell layer; C = osteosclereid layer; D = parenchyma layer. Bar = 20 μm.

The seed coat of *B. basaltica* and *B. bituminosa* has the typical pattern of legumes with hard seeds. In both species, the outermost layer consists of a uniseriate closely packed epidermis of macrosclereids (i.e., palisade cell layer), with a mean thickness of 100 μm ± 3 SE (standard error) in *B. basaltica* and 80 μm ± 4 SE in *B. bituminosa*. The next cell layer consists of osteosclereids, i.e., dead thick-walled cells, hourglass shaped forming large inter-cellular air spaces. The last layer is a parenchyma composed of several layers of flattened cells (Figure 2a,b).

At the end of the imbibition test, intact BD pods of *B. basaltica* and *B. bituminosa* had imbibed an amount of water equal to 23 and 40% of their initial dry mass, respectively. Moreover, during the test, seeds from imbibed pods (3% of the seeds for *B. basaltica* and 5% for *B. bituminosa*) germinated. All the remaining pods showed to be non-imbibed, due to the impermeability of the seed coat (PY occurrence), and seeds proved to be viable after the following scarification and cut-test.

### 2.2. Effect of Dormancy-Breaking Treatments on Germination Behavior

**Figure 3:**
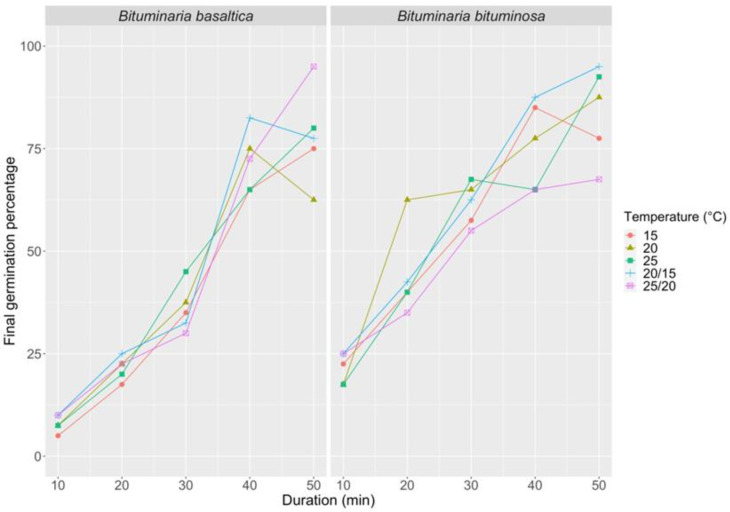
Final germination percentage at different incubation temperatures (15, 20, 25, 20/15, 25/20 °C, 12/12 h photoperiod and thermoperiod) in *B. basaltica* and *B. bituminosa,* after different immersion times (10, 20, 30, 40, 50 min treatment duration) in H_2_SO_4_.

Mechanical, thermal and chemical treatments were evaluated for their relative effectiveness in breaking dormancy in seeds from intact BD pods. Our results showed that the effectiveness of a chemical treatment with H_2_SO_4_ increased significantly with increasing immersion time (*p* < 0.0001 for both species; Supplementary data Table S1). The best Final Germination Percentage (FGP) was observed after 40 and 50 min immersion for both *B. basaltica* (FGP ranging from 63% to 95%) and *B. bituminosa* (FGP ranging from 65% to 95%). With these immersion times, FGPs were similar for the two species, while with 10–30 min immersion FGPs were higher in the latter species (Figure 3). Neither a significant effect of incubation temperature on FGP nor a significant interaction between treatment duration and temperature was observed in each species (Supplementary data Table S1). All ungerminated seeds proved to be viable after chemical treatment, after mechanical scarification and following cut-test.

**Figure 4:**
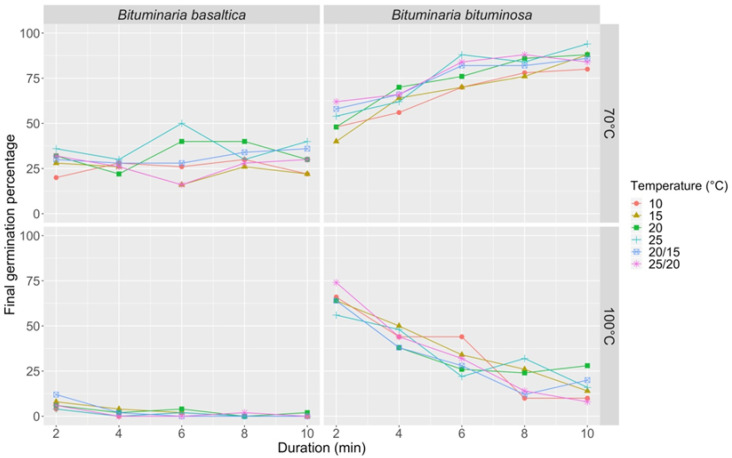
Final germination percentage at different incubation temperatures (10, 15, 20, 25, 20/15, 25/20 °C) in *B. basaltica* and *B. bituminosa* after different immersion times (2, 4, 6, 8, 10 min treatment duration) in hot constant water (70 and 100 °C).

The first series of thermal treatments (70 and 100 °C hot constant water; 10, 15, 20, 25, 20/15, and 25/20 °C incubation temperature) showed a significant effect of water temperature on FGP (*p* < 0.0001 for both species; Supplementary data Table S2). The 70 °C treatment produced a small effect in *B. basaltica*, bringing to a FGP of 50% (6 min immersion time and 25 °C incubation temperature; Figure 4) and did not produce any damage to the ungerminated fraction, as shown by mechanical scarification and cut-test. In the same species, the 100 °C treatment did not improve FGP (max FGP = 12%; Figure 4), and produced lethal effects on the whole ungerminated fraction. In *B. bituminosa*, the two treatments (70 and 100 °C) determined opposite trends at increasing immersion time: at 70 °C, FGP increased from 40 to 94%, while at 100 °C, FGP decreased from 74 to 8%, respectively (Figure 4). The 100 °C hot water treatment determined lethal effects on the ungerminated fraction also in *B. bituminosa*. In relation to incubation temperature, a significant effect on FGP was detected in *B. basaltica* (*p* = 0.0014), while no effect was observed in *B. bituminosa*.

**Figure 5:**
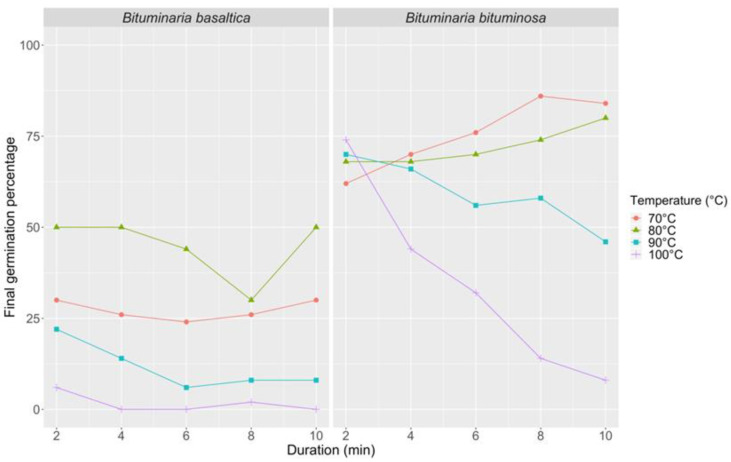
Final germination percentage at 25/20 °C incubation temperature in *B. basaltica* and *B. bituminosa* after different immersion times (2, 4, 6, 8, 10 min treatment duration) in hot constant water (70, 80, 90, and 100 °C).

The second series of thermal treatments (70, 80, 90, and 100 °C hot constant water; 25/20 °C incubation temperature) confirmed the water temperature effect on FGP in both species (Figure 5), as well as a significant interaction between water temperature and immersion time (*p* = 0.0008 for *B. basaltica* and *p* < 0.0001 for *B. bituminosa*; Supplementary data Table S3). Treatments with hot water were little effective with *B. basaltica*, where FGP values never exceeded 50%. In *B. bituminosa*, FGP increased with immersion time for treatments with 70 and 80 °C hot water, up to 86 and 80%, respectively. Otherwise, FGP decreased with immersion time for treatments with 90 and 100 °C hot water. These latter treatments produced lethal effects and most of the ungerminated seeds were unviable after the tests.

**Figure 6:**
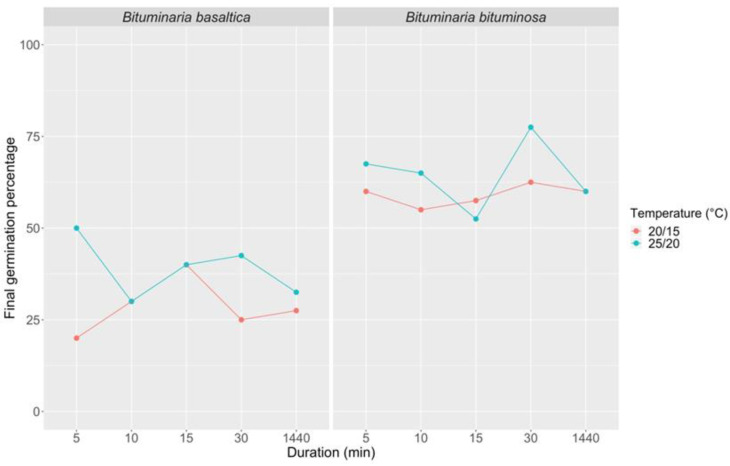
Final germination percentage at 20/15 and 25/20 °C incubation temperatures in *B. basaltica* and *B. bituminosa* after different immersion times (5, 10, 15, 30, 1440 min treatment duration) in boiling water gradually cooling up to room temperature.

Thermal scarification in boiling water, allowed to cool gradually to room temperature for different immersion times (from 5 to 1440 min treatment duration), was quite effective in promoting *B. bituminosa* germination (max FGP = 77%, 30 min treatment duration and 25/20 °C incubation temperature; Figure 6). These treatments did not increased FGP in *B. basaltica* over 50% (5 min treatment duration; 25/20 °C incubation temperature; Figure 6; Supplementary data Table S4).

**Figure 7:**
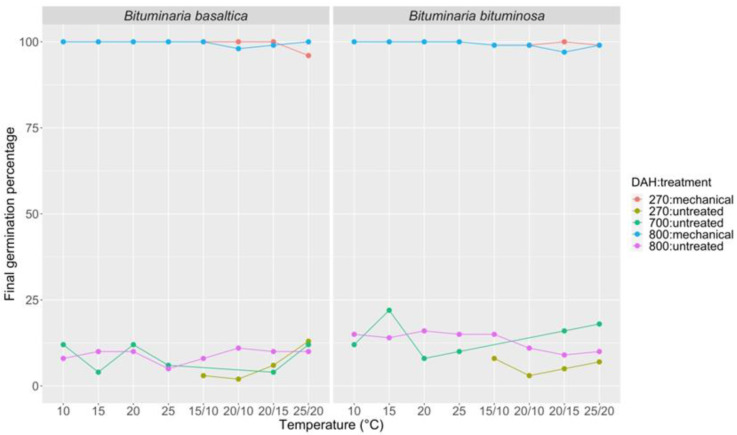
Final germination percentage at different incubation temperatures (10, 15, 20, 25, 15/10, 20/10, 20/15, 25/20 °C) for *B. basaltica* and *B. bituminosa* seeds at 270, 700, and 800 days after harvesting (DAH); pods untreated and after mechanical scarification.

Mechanical scarification produced optimal results on seed germination of both species, compared to control conditions, with FGP reaching about 100%, regardless of seed age (Figure 7; Supplementary data Table S5). Seeds of both species kept viable for over two years.

**Figure 8:**
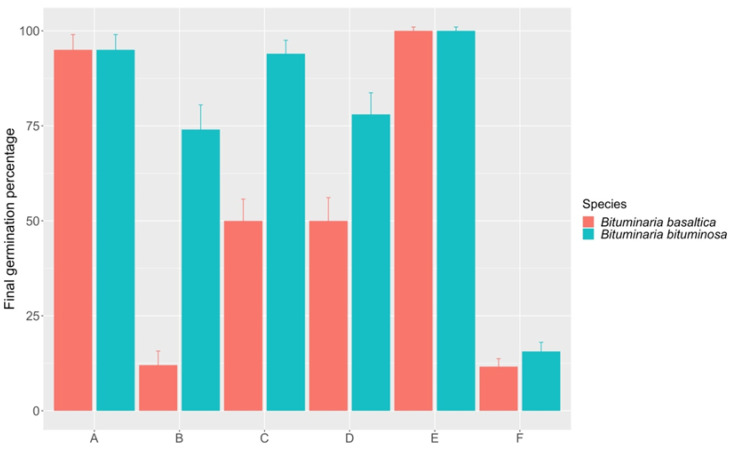
Summary of the most effective dormancy-breaking treatments. A: chemical treatment (50 min immersion time) and incubation temperature at 25/20 °C (*B. basaltica*) and 20/15 °C (*B. bituminosa*); B: thermal treatment at 100 °C (2 min immersion time) and incubation temperature at 20/15 °C (*B. basaltica*) and 25/20 °C (*B. bituminosa*); C: thermal treatment at 70 °C (6 min immersion time for *B. basaltica* and 10 min for *B. bituminosa*) and incubation temperature at 25 °C (both species); D: thermal treatment at 100 °C with gradual cooling (5 min immersion time for *B. basaltica* and 30 min for *B. bituminosa*) and incubation temperature at 25/20 °C for both species; E: mechanical treatment; F: untreated. Vertical bars represent standard errors.

In summary, considering all treatments, mechanical and chemical treatments proved to be the most effective scarification methods, resulting in complete or almost complete germination in both species (Figure 8). Among chemical treatments, the best germination performance (95%) was obtained at the highest immersion time value (i.e., 50 min), for both, *B. basaltica* and *B. bituminosa*. Thermal treatments resulted in high FGP levels only in *B. bituminosa* (94%, 10 min—70 °C hot constant water; 77%, 30 min—boiling water gradually cooling at room temperature; 74%, 2 min —100 °C hot constant water), while showed poorer performances (50%, both 10 min—70 °C hot constant water and 5 min—boiling water gradually cooling) or even a detrimental effect in *B. basaltica* (12%, 2 min—100 °C hot constant water).

### 2.3. Pod Maturity Stages and Seed Germination Behavior over Time

**Figure 9:**
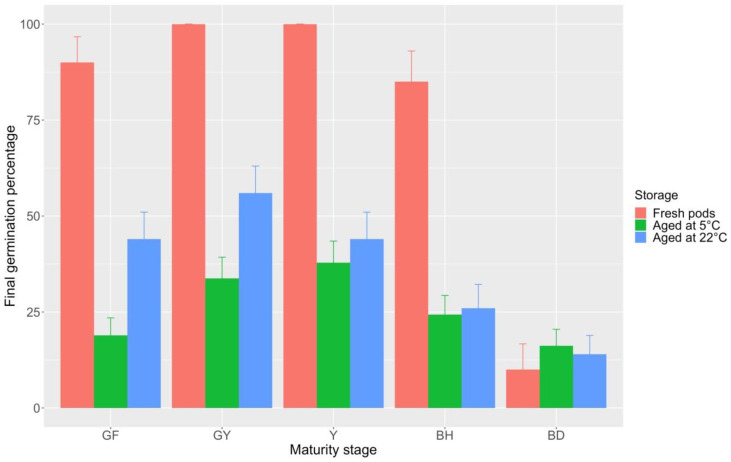
Final germination percentage at 20 °C incubation temperature for *B. bituminosa* seeds at different pod maturity stages, at harvest time (fresh) and after 420 days of storage at 5 ± 1 and 22 ± 2 °C. Vertical bars represent standard errors. GF = green full; GY = green yellow; Y = yellow; BH = brown hydrated; BD = brown dehydrated.

Seeds at the different pod maturity stages of *B. bituminosa*, except brown dehydrated (BD) ones, showed very high FGP values at harvest time (20 °C incubation temperature; Figure 9). Specifically, seeds from both green full (GF) pods and brown hydrated (BH) pods were characterized by germination values of 90 and 85%, respectively, while those from green-yellow (GY) and yellow (Y) pods reached full germination. Finally, germination capability of seeds from BD pods did not exceed 10%, a pattern that can be attributed to coat-imposed dormancy, as indicated by the full recovery effect of pod scarification.

Germination behavior of seeds at the different pod maturity stages was assessed also after a storage period of 420 days at two different temperature conditions (5 °C ± 1 °C and 22 ± 2 °C). Germination performance of seeds from BD pods was not affected by storage time, which determined a FGP decrease of about 50% in the seeds from the remaining maturity stages instead (Figure 9). Specifically, the drastic decrease was independent from storage conditions in seeds from BH (FGP = 30% for 5 ± 1 °C storage condition; FGP = 26% for 22 ± 2 °C storage condition) and Y pods (FGP = 44% for 5 ± 1 °C storage condition; FGP = 47% for 22 ± 2 °C storage condition). Conversely, a storage type effect was observed in the earlier maturity stages, i.e., GY (FGP = 33% for 5 ± 1 °C storage condition; FGP = 56% for 22 ± 2 °C storage condition) and GF pods (FGP = 28% for 5 ± 1 °C storage condition; FGP = 44% for 22 ± 2 °C storage condition; Figure 9). Checking for seed viability by needle pin showed that ungerminated seeds were viable.

**Figure 10:**
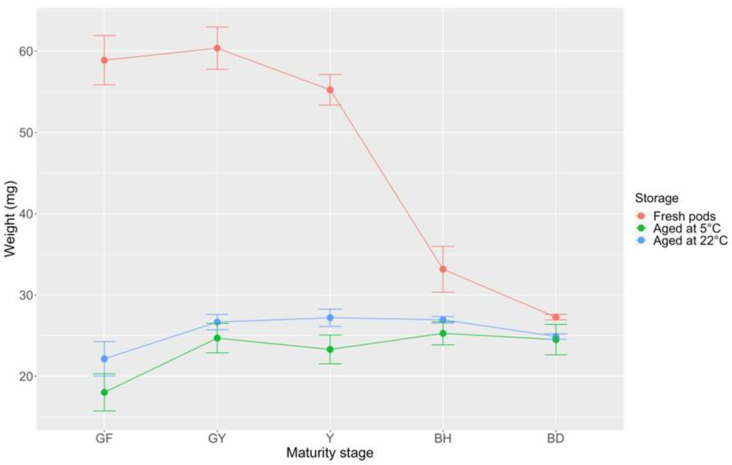
Mass of *B. bituminosa* fresh and stored pods at different maturity stages (420 days at 5 ± 1 °C and 22 ± 2 °C). Vertical bars represent standard errors. GF = green full; GY = green yellow; Y = yellow; BH = brown hydrated; BD = brown dehydrated.

*B. bituminosa* fresh pods at different maturity stages showed highly significant differences in pod mass, ranging from 60.36 mg (GY) to 27.27 mg (BD) (Figure 10). Specifically, a steep mass decrease was detected between Y (55.24 mg) and BH fresh pods (33.17 mg; Figure 10), due to their moisture content drop. However, storage periods of 420 days at both 5 ± 1 °C and 22 ± 2 °C neutralized these relevant differences, except for GF pods, which showed lower mass values than other maturation stages, at both storage conditions. Furthermore, mass uniformity across GY, Y, and BH stored pods, regardless of storage conditions, suggests that dry matter accumulation had been stopped in correspondence with the GY maturity stage, at which pod drying process, conversely, had been started. Therefore, it is arguable that BH and BD pods also had approximately the same constant dry matter content of Y and GY pods. Both BD and BH pods have reached full maturity but BD pods have also achieved an additional important physiological goal along pod development process, i.e., the ‘maturation drying’ (desiccation), with a general reduction in water content. At this stage, *B. bituminosa* seeds have acquired dormancy, being no more able to germinate, due to seed coat impermeabilization (PY).

**Figure 11:**
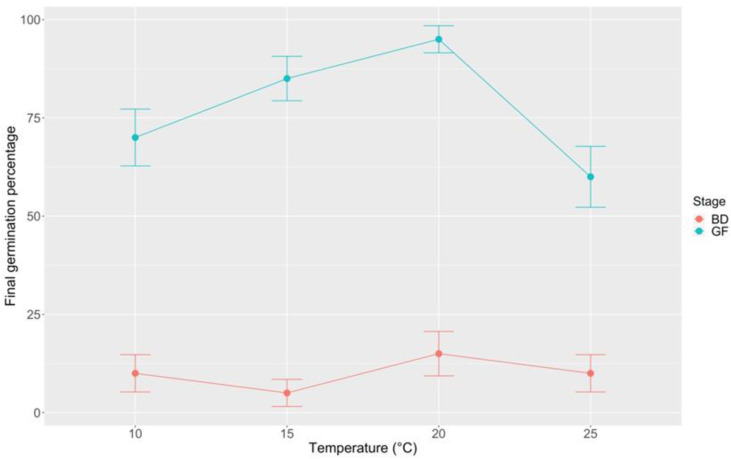
Final germination percentage at different incubation temperatures (10, 15, 20, 25 °C) for *B. bituminosa* seeds from GF and BD pods after 180 days of storage at 5 ± 1 °C. Vertical bars represent standard errors. BD = brown dehydrated; GF = green full.

Germination assays at constant incubation temperatures (10, 15, 20, and 25 °C) after 180 days of storage at 5 ± 1 °C showed that *B. bituminosa* seeds from GF pods retained high germination capability, which increased from 70 to 95% in the range 10–20 °C, while decreased at 25 °C (Figure 11). Accordingly to previous data (Figure 9), seeds from BD pods exhibited very low germination values under all temperature regimes (FGP ranging from 5 to 15%), resulting viable after mechanical scarification.

**Figure 12:**
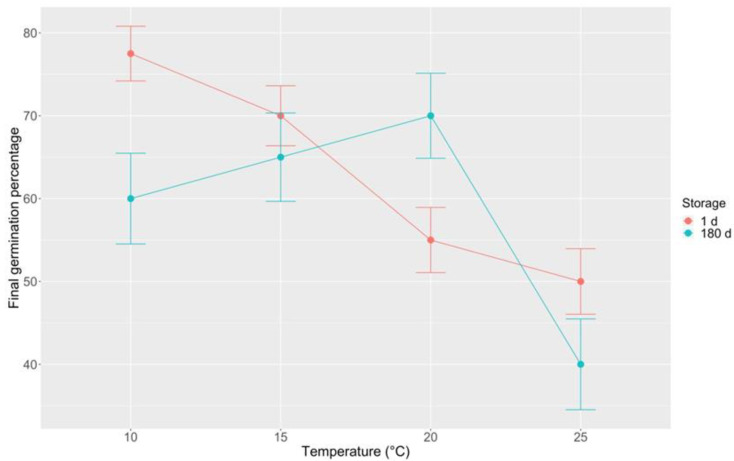
Final germination percentage at different incubation temperatures (10, 15, 20, 25 °C) for *B. basaltica* seeds from fresh (1 day) and stored (after 180 days at 5 ± 1 °C) GF pods. GF = green full. Vertical bars represent standard errors.

Additional assays on GF pods of *B. basaltica* have revealed some similarities with *B. bituminosa* germination behavior. Indeed, seeds from fresh GF pods showed rather high FGP values, and exhibited a preference for lower temperatures. A clear negative germination trend at increasing incubation temperature was observed in *B. basaltica* (FGP from 77 to 50% in the range 10–25 °C; FGP = 56% at 20 °C; Figure 12), differently from *B. bituminosa* (FGP = 90% at 20 °C incubation temperature; Figure 11). Like this species, *B. basaltica* seeds from GF pods retained a high germination capability after 180 days of storage at 5 ± 1 °C, showing a positive germination trend with increasing incubation temperature, in the range 10–20 °C, and a FGP decrease at 25 °C (max FGP = 70% at 20 °C; Figure 12).

## 3. Discussion

Knowledge on seed germination requirements is crucial to assess the real potential of wild genetic resources to be exploited within agricultural systems [46,47]. This is particularly important for legumes, where the role of seed coat in imposing PY has been widely recognized [6,7,48]. In this study, we analyzed pod morphometric traits, seed coat anatomy and germination behavior of two Mediterranean species within the promising multiple-use *Bituminaria* genus: the widespread *B. bituminosa* and the local insular point endemic *B. basaltica*. As the first step, we took pods from natural populations in different wild environments, reflecting the geographical distribution of these two species. Therefore, the observed differences may reflect not only genetic traits, but also the environmental effects; based on our results, future ex situ studies should be planned to compare these two species under a common environment, so that genetic effects may be more clearly assessed.

Overall, we found differences in morpho-anatomical traits and a promoting effect of all the scarification treatments on germinability, with mechanical and chemical methods being the most effective for both species. We also found that seeds at early maturity pod stages showed higher germination capacity and that mature seeds retain germinability for long time after harvesting.

Our germination tests showed that untreated fresh mature seeds (BD pods) could germinate only at very low FGP values in *B bituminosa* (Figure 9). Nevertheless, when mechanically scarified, these seeds showed an almost full germination, hence strongly suggesting the existence of a PY mechanism in the investigated species. Accordingly, imbibition tests conducted on fresh mature BD pods resulted in a very limited water absorption in both study species, and the observed mass increasing was mainly due to water-uptake by the minor ‘soft seed fraction’. Further, the seed coat in both species has a water-impermeable palisade layer, a tissue typically found in true seed coats or pericarps of the germination unit of species with PY [7].

Previous studies conducted on seed germination in the *B. bituminosa* complex showed contrasting results. For instance, high mean germination percentage without any scarification treatment was reported in *B. bituminosa* var. *bituminosa* from Calnegre, Spain (about 70%; [39]) and from Morocco (90%; [43]). In *B. bituminosa* var. *albomarginata* from Canary Islands evidence of non-deep physiological dormancy, which lasted around three months, was also detected [40]. Our findings expand the knowledge of germination patterns in this species complex in an under-investigated area and are consistent with PY, even because seeds remained dormant even after a three month-period and over (until 800 days after harvesting).

Our results suggest that PY establishes during seed maturation in both study species, as already reported for other legumes [49,50]. Specifically, in *B. bituminosa* the availability of pods at five different maturity stages allowed us to observe that seeds from four of them (GF, GY, Y, and BH) showed very high FGP values, as previously observed by Duguma et al. (1988) [51] in *Leucaena leucocephala*, but differently from what Samarah et al. (2003) [52] highlighted in *Vicia sativa*. In our study, even seeds from fresh GF pods were readily able to germinate, as non-dormant, while PY established between the two latest maturity stages (i.e., BH and BD pods), when pod color had already changed to brown and a steep seed mass decrease had occurred. These findings are consistent with those obtained in other pasture Mediterranean legumes [11,18,53].

Seeds of both *B. basaltica* and *B. bituminosa* at GF pod maturity stage retained high germination ability (FGP = 95 and 70% at 20 °C, respectively), at least after a storage period of 180 days at 5 °C (Figure 11 and Figure 12). Suitable plant genetic resources for multiple application purposes have to provide viable and readily germinable seeds at sowing time. Moreover, it should be possible to plan sowing when full-field conditions are suitable for seed germination and seedling establishment. Our findings on storage behavior in the study species are consistent with the proposal of a seed management protocol that, in Mediterranean habitats, could provide for an early harvest of GF pods in late spring, followed by a cool temperature storage for about four-five months and, finally, an autumn sowing at the onset of the wet season. This protocol would take advantage of free germination of seeds at GF pod maturity stage as a major trait for supporting the ‘pre-cultivation/domestication’ process of wild *Bituminaria* species, reducing also the risk of seed losses, which may be caused by natural pod shedding in case of delayed harvesting. Early harvesting influences seed germination and dormancy and the effect is species-dependent [52]. Therefore, further studies are needed to test the germination behavior of seeds at the other pod maturity stages after 180 day-cool storage, in order to verify eventual benefits of using them versus GF pods. Indeed, if other early maturity stages could be used in a similar fashion, the cue of the coexistence of pods at different maturity stages within flower heads would be overcome. Moreover, the effect of storage times longer than 180 days should also be investigated, as non-dormant seed fraction decreased markedly at 420 day-storage. Finally, a further issue to be focused, under a cultivation perspective, could be the vigor of seedlings from different maturity stage pods.

As an alternative to the above-mentioned protocol, BD pods could be used for sowing, provided that suitable dormancy-breaking treatments are adopted. Indeed, hardseedeness in legumes may be artificially overcome by a variety of treatments [17,54]. Furthermore, our study found that BD pods maintain their viability for long time (up to 800 days), proving to be an effective long-term seed source. Hence, different types of treatments were tested, in order to find the most efficient, i.e., those which could provide the highest FGP values (without embryo damage), being convenient to use for large amount of pods as well. Overall, mechanical, chemical and thermal treatments provided a positive effect on seed germination, compared to control conditions. However, not all treatments had the same effectiveness and, not less important, the two study species showed different sensitivity to them.

Chemical and mechanical scarification proved to be the most effective methods, causing full or quite full germination in both species (Figure 3 and Figure 7), regardless of incubation temperature but depending on immersion time in H_2_SO_4_. Indeed, as regards chemical treatments, the greatest germination performance (95%) in both species was obtained with the highest immersion time value (i.e., 50 min). Despite of their high effectiveness in overcoming PY, neither mechanical nor chemical treatments should be of practical value for large-scale application, due to their specific disadvantages. Mechanical scarification, particularly when conducted by a needle pin as in our study, should be a very time-consuming method when large seed quantities are needed, and the alternative use of some industrial equipment should be checked for seed loss due to embryo injury and/or reduced seedling vigor. On the other hand, chemicals may be difficult to obtain and hazardous to store and handle. Our results point to thermal treatments as the best large-scale scarification methods, resulting in high FGP levels in *B. bituminosa* (94%, 10 min—70 °C hot constant water; 77%, 30 min—boiling water gradually cooling at room temperature) and in good ‘trade-off’ FGP levels in *B. basaltica* (50%, both 10 min—70 °C hot constant water and 5 min—boiling water gradually cooling). Hence, treatments with gradually cooling boiling water could also be taken into account under a large-scale application perspective, as they proved to be relatively safer and more user-friendly.

Considering the differences between species in the sensitivity to scarification methods, *B. basaltica* showed a general lower susceptibility to all scarification treatments, except for the mechanical method. Specifically, we observed a lower seed germination response both to thermal treatments and to chemical scarification, in the range 10–30 min immersion time. Lower sensitivity to dormancy-breaking treatments in *B. basaltica* might be related to the greater thickness of the palisade cell layer observed in this species, which could affect the lower permeability level of the seed coat. Some evidence that seed coat thickness is associated with PY and/or PY release exists [55,56,57]. However, other studies did not find a direct relationship between absolute seed coat thickness and dormancy [58]. Moreover, seed coat lower permeability could be related to testa’s chemical composition, since greater amount of components that repel water, such as polyphenols, lignins, and tannins, was observed already in *Medicago truncatula*, *Pisum sativum*, *Medicago sativa* L., and in *Trifolium repens* L. [56,59,60,61]. Further, the only involvement of the thickness of the palisade cell layer should not explain the more marked detrimental effect of thermal treatments observed in *B. basaltica*. Therefore, it is arguable that other specific anatomical structures are involved in the response to dormancy-breaking treatments. Indeed, a great variety of ‘water gaps’ have been already detected, and they may differently be affected by different types of dormancy-breaking treatments [62], with the lens and the hilar slit being the structures described in Papilionoideae so far [63]. In this perspective, scarification by needle pin broke PY in both the species under investigation, through by-passing whatever specific seed coat peculiarity, and producing a clear water entry in the seed coat, which explains the lack of difference between the response of the two species.

In addition to a thicker palisade layer of the seed testa, *B. basaltica* showed also smaller and lighter pods compared to *B. bituminosa*. This circumstance gives additional insights on the relation between seed size and seed dormancy. Indeed, some authors highlighted that the proportion of dormant seeds increases with seed size decreasing [64,65,66], and that large seeds become sensitive to environmental cues that break PY earlier than small ones [67]. However, based on available data, our study does not allow any conclusive observation for the study species.

## 4. Materials and Methods

### 4.1. Study Species and Sampling Strategy

**Figure 13:**
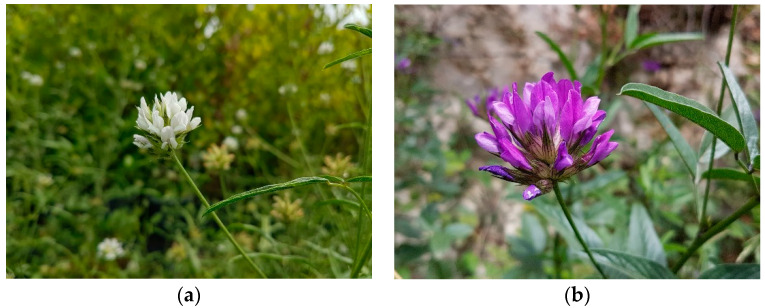
(**a**) Bituminaria basaltica; (**b**) Bituminaria bituminosa.

*Bituminaria basaltica* Miniss., C. Brullo, Brullo, Giusso and Sciandr. is a herbaceous perennial species endemic to the Filicudi island (Aeolian Archipelago—Tyrrhenian Islands, Italy; Figure 13a). It grows in steppe grasslands, abandoned fields, and roadsides, on volcanic substrates, at altitudes between 20 and 280 m a.s.l., in the dry upper thermo-Mediterranean bioclimatic plane [45].

*Bituminaria bituminosa* (L.) C.H. Stirt. is a perennial legume species widely distributed in the Mediterranean Basin and Macaronesia (Figure 13b). It is considered as a potential new pasture for arid and semi-arid Mediterranean ecosystems [3]. The interest for this taxon is linked to its remarkable ecological plasticity (deep-rootedness, drought tolerance), being able to live both in very arid coastal habitats (<250 mm/year) and in extremely rainy mountain stations. The nature of the substrates in which the species is found is also very variable both from the particle size and from the chemical point of views [3].

Mature monospermic pods (brown dehydrated = BD, containing seeds at mass maturity, i.e., with maximum dry weight) of the two species were collected in 2015 (Table 1), at the time of their natural dispersion. In order to acquire a reliable representation of the genetic diversity, pods were randomly collected from over 100 individuals per population.

**Figure 14:**
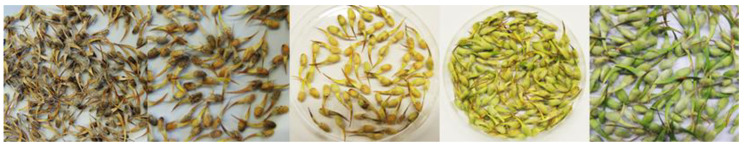
Pod maturity stages in *B. bituminosa*. From left to right: BD = brown dehydrated, BH = brown hydrated, Y = yellow, GY = green yellow and GF = green full pods.

In addition, pods of *B. bituminosa* were harvested from the same mother plants at the following maturity stages (Figure 14): green full (GF), green-yellow (GY), yellow (Y), brown hydrated (BH). Those of *B. basaltica* were collected only at two maturity stages: green full (GF) and brown dehydrated (BD), as the other stages were not as clearly recognizable as in the former species. The color refers to the ripe legume wall since the seed is tightly fixed in the pod. Specifically, with ‘green full pod’ we refer to a fully expanded green pod, where the unique seed fills the pod cavity and shows a complete and well-shaped embryo after cut-test.

Plant material was transferred to the germplasm bank laboratories of the Department of Biological, Geological and Environmental Sciences (University of Catania).

### 4.2. Morphometric Analysis and Seed Coat Anatomical Structure

Mean BD pod mass was estimated at 30 days after harvest for both species, based on four replicates of 50 pods, by means of an electronic balance (0.0001 g precision—Mettler Toledo AE-50, Zurich, Switzerland). Digital images of 10 BD pods were acquired using an Olympus SZX 12 stereo microscope equipped with an Olympus DP70 digital camera (Olympus Optical, Tokyo, Japan). Captured digital images were then analyzed with the software Analysis^®^ Five (Olympus Soft Imaging Solutions, Münster, Germany). Six descriptive morphometric parameters were measured for each pod: width, height, area, perimeter, primary axis of best fitting ellipse (Max X), and secondary axis of best fitting ellipse (Max Y).

Seed coat anatomy was observed for each species on BD pods, which were preliminarily immersed in distilled water for 24 h. Samples without embedding were fixed on a specimen holder. Then fresh sections were obtained using the Leica RM2125 RTS rotary microtome (Leica Biosystems, Nussloch, Germany) and were directly observed under the Olympus BX 40 optical microscope (Olympus Optical, Tokyo, Japan), equipped with the Olympus DP 70 digital camera. Measurements were taken on three pods for each species.

To investigate seed PY, a water imbibition test was conducted in both the study species at 270 days after harvest. Initial pod mass (W_o_) was determined for four replicates of 50 intact BD pods each (indehiscent fruits: seeds + pericarp). Replicates were incubated at room temperature (22 ± 2 °C) in 140 mm diameter Petri dishes on three sheets of filter paper moistened with 25 mL distilled water. The pods were blotted dry between paper towels, to remove any surface water, and water imbibed by the pods was determined after 24, 48, and 72 h (i.e., until seeds within each replicate begun to germinate). Percentage water uptake was calculated as pod mass increase based on initial pod mass (*W_o_*):

%*W_s_* = [(*W_i_* − *W_o_*)/*W_o_*] × 100 (1)

where *W_s_* = increase in mass of pods, *W_i_* = mass of pods after a given interval of imbibition, and *W_o_* = initial pod mass.

After the imbibition test, only the non-imbibed pods were scarified by needle pin and incubated at 20 °C for 15 days. Specifically, mechanical scarification was achieved by pricking pods, far from the radical pole, by means of a 0.35 mm diameter needle.

### 4.3. Seed Dormancy-Breaking Treatments and Germination Assessment

*B. basaltica* and *B. bituminosa* show indehiscent legumes, and, therefore, pods were used as germination units in laboratory tests. Nevertheless, the word seed is also employed in the text when referring to germination and dormancy, since the last two term are intrinsically linked to seed behavior. Mechanical, thermal and chemical treatments were compared in terms of their relative effectiveness in breaking seed dormancy in intact BD pods. Mechanical scarification was achieved by needle pin, as previously described. Thermal scarification was achieved by soaking pods both in hot distilled water at constant temperature (70, 80, 90, 100 °C) and in boiling water gradually cooling to room temperature (22 ± 2 °C) for different times (from 5 to 1440 min). Chemical scarification was accomplished by soaking pods in H_2_SO_4_ (98%), and then washing them thoroughly in sterile distilled water before germination tests. Intact pods without any treatments were used as controls (Table 2).

Germination experiments were performed in controlled-temperature growth chambers (Sanyo—model MLR-351H, Tokyo, Japan), placing pods in Petri dishes on three layers of filter papers moistened with 5 mL distilled water. For each germination assay, four replicates of 25 randomly selected pods were used. All Petri dishes were sealed using Parafilm M^®^ to avoid moisture loss, adding water as needed to keep an adequate moisture level. Germination tests were performed on more than 15,000 pods for each species. In detail, germination assays of non-scarified (control) and scarified pods were tested across a range of constant temperature (10, 15, 20, 25 °C) and fluctuating temperatures (15/10, 20/10, 20/15, 25/20 °C), in light/dark conditions (L/D) with a 12/12 h photoperiod. Fluctuating temperature regimes were selected considering the values that are normally experienced by pods in natural conditions during the germination season near to the soil surface (from autumn to early spring). In the experiments with alternating temperature, the exposure to light coincided with the highest temperature to replicate day/night conditions. Germination was investigated for fresh and laboratory-stored pods.

Germinations were monitored daily, and pods with germinated seeds were counted and removed from Petri dishes. Germination was considered as achieved when the radicle protrusion was about 2 mm. Experiments were continued for 30 days. At the end of each germination test, ungerminated seeds were reincubated after pod scarification by needle pin, for a further 15-day period to be checked for viability. Finally, a cut-test was applied to the eventually ungerminated seeds. Seeds with a plump, firm, and white embryo were considered as viable. The final germination percentage (FGP) was calculated on the basis of the total number of filled seeds.

### 4.4. Effect of Pod Maturity Stage and Storage Conditions on Germination Behavior

In order to assess the effect of pod maturity stage on seed germination and dormancy establishment, germination tests were performed immediately after pod collection (20 °C, L/D—12/12 h photoperiod), on each of the above mentioned stages (GF, GY, Y, BH, BD) for *B. bituminosa* and only on GF stage for *B. basaltica*. Additional tests were performed after longer storage times (180 and 420 days) and at different storage temperatures (22 °C ± 2 °C and 5 °C ± 1 °C; Table 2). To monitor pod mass changes over time, mean pod mass was estimated for each pod maturity stage (five replicates of 10 pods) only in *B. bituminosa*, soon after pod collection and after a storage period of 420 days at both 5 ± 1 °C and 22 ± 2 °C.

### 4.5. Data Analysis

Morphometric data were analyzed by using heteroscedastic Welch tests. The counts of germinated seeds were analyzed by using generalized linear models, with binomial error and logit link. The FGPs were derived from parameter estimates, together with ‘delta’ standard errors [68] and were reported in Figure 8, Figure 9, Figure 10 and Figure 11, and in Supplementary Data Tables S1–S5. All analyses were performed by using the R statistical environment [69].

## 5. Conclusions

In conclusion, our results showed that PY may reduce the germination capability in two populations of the *Bituminaria* complex, which had not yet been investigated (one of which, *B. basaltica*, recently described as a new species). Besides, our results also provided technical solutions to overcome PY by showing the best practices for seed collection-time, storage and dormancy breaking. Differences emerged between *B. basaltica* and *B. bituminosa*, in both their pod traits and responses to dormancy-breaking treatments, which support future specific trials exploring the actual anatomical structures ad hoc involved in dormancy regulation. Overall, our data contributed to the background knowledge needed for planning the introduction of *Bituminaria* species within agricultural systems. The future characterization of additional *Bituminaria* genetic resources from other Mediterranean populations will have remarkable importance.

## Figures and Tables

**Table 1:** Sampling locations for *Bituminaria basaltica* and *B. bituminosa*.

Species	Date	Site	Elevation m a.s.l.	Coordinates (WGS84)	
				Latitude	Longitude
*Bituminaria basaltica*	July 2015	Filicudi (Messina, Italy)	283	38° 34′ 07.70”	14° 34′ 14.99”
*Bituminaria bituminosa*	July 2015	Fiumefreddo di Sicilia (Catania, Italy)	123	37° 48′ 12.56”	15° 11′ 51.56”

**Table 2:** Experimental design of the germination tests conducted in the laboratory.

Treatment		Maturity Stage	Seed Age	Storage Condition	Immersion Time	Incubation Temperature
Untreated—*B. basaltica*		GF	1	-	-	10, 15, 20, 25 °C
		GF	180	5 ± 1 °C	-	10, 15, 20, 25 °C
Untreated—*B. bituminosa*		BD	1	-	-	20 °C
		BH	1	-	-	20 °C
		Y	1	-	-	20 °C
		GY	1	-	-	20 °C
		GF	1	-	-	20 °C
		BD	420	5 ± 1 °C	-	20 °C
		BH	420	5 ± 1 °C	-	20 °C
		Y	420	5 ± 1 °C	-	20 °C
		GY	420	5 ± 1 °C	-	20 °C
		GF	420	5 ± 1 °C	-	20 °C
		BD	420	22 ± 2 °C	-	20 °C
		BH	420	22 ± 2 °C	-	20 °C
		Y	420	22 ± 2 °C	-	20 °C
		GY	420	22 ± 2 °C	-	20 °C
		GF	420	22 ± 2 °C	-	20 °C
		GF	180	5 ± 1 °C	-	10, 15, 20, 25 °C
		BD	180	5 ± 1 °C	-	10, 15, 20, 25 °C
Mechanical	needle pin	BD	270	22 ± 2 °C	-	15/10, 20/10, 20/15 25/20 °C
		BD	800	22 ± 2 °C	-	15/10, 20/10, 20/15, 25/20 °C 10, 15, 20, 25 °C
Thermal	boiling water gradually cooling	BD	300	22 ± 2 °C	5, 10, 15, 30, 1440 min	20/15, 25/20 °C
	70, 100 °C hot constant water	BD	700	22 ± 2 °C	2, 4, 6, 8, 10 min	20/15, 25/20 °C 10, 15, 20, 25 °C
	70, 80, 90, 100 °C hot constant water	BD	840	22 ± 2 °C	2, 4, 6, 8, 10 min	25/20 °C
Chemical	H_2_SO_4_ (98%)	BD	450	22 ± 2 °C	10, 20, 30, 40, 50 min	20/15, 25/20 °C 15, 20, 25 °C
Control		BD	270	22 ± 2 °C	-	15/10, 20/10, 20/15, 25/20 °C
		BD	700	22 ± 2 °C	-	20/15, 25/20 °C 10, 15, 20, 25 °C
		BD	800	22 ± 2 °C	-	15/10, 20/10, 20/15, 25/20 °C 10, 15, 20, 25 °C

## Supplementary Materials

The following are available online at https://www.mdpi.com/2223-7747/9/9/1110/s1. Table S1: Final germination percentage (FGP) with standard error (*SE*) at different incubation temperatures (15, 20, 25, 20/15, 25/20 °C) in *B. basaltica* and *B. bituminosa*, after different immersion times in H2SO4. Percentages were derived by back-transformation of model parameters, following a GLM fit (see materials and methods for details), Table S2: Final germination percentage (FGP) with standard error (*SE*) at different incubation temperatures (10, 15, 20, 25, 20/15, 25/20 °C) in *B. basaltica* and *B. bituminosa*, after different immersion times in hot constant water (100 and 70 °C). Percentages were derived by back-transformation of model parameters, following a GLM fit (see materials and methods for details), Table S3: Final germination percentage (FGP) with standard error (*SE*) in *B. basaltica* and *B. bituminosa* at 25/20 °C incubation temperature, after different immersion times in hot constant water (70, 80, 90 and 100 °C). Percentages were derived by back-transformation of model parameters, following a GLM fit (see materials and methods for details), Table S4: Final germination percentage (FGP) with standard error (*SE*) in *B. basaltica* and *B. bituminosa* at 20/15 and 25/20 °C incubation temperatures, after different immersion times in boiling water gradually cooling up to room temperature. Percentages were derived by back-transformation of model parameters, following a GLM fit (see materials and methods for details), Table S5: Final germination percentage (FGP) with standard error (*SE*) in *B. basaltica* and *B. bituminosa* at different incubation temperatures (10, 15, 20, 25, 15/10, 20/10, 20/15, 25/20 °C); pods untreated and after mechanical scarification. Percentages were derived by back-transformation of model parameters, following a GLM fit (see materials and methods for details).

## References

[1] Maxted N., Bennett S.J., Maxted N., Bennett S.J. (2001). Legume diversity in the Mediterranean region. Plant Genetic Resources of Legumes in the Mediterranean.

[2] Brígido C., Menéndez E., Paço A., Glick B.R., Belo A., Félix M.R., Oliveira S., Carvalho M. (2019). Mediterranean native leguminous plants: A reservoir of endophytic bacteria with potential to enhance chickpea growth under stress conditions. Microorganisms.

[3] Pazos-Navarro M., Dabauza M., Correal E., Walker D., del Río J.A., Ortuño A., Méndez P., Santos A., Ríos S., Martínez-Frances V., Satou H., Nakamura R. (2013). Legumes for grazing and health: The Case of *Bituminaria Bituminosa*. Legumes: Types, Nutritional Composition and Health Benefits.

[4] Melis R.A.M., Pecetti L., Annicchiarico P., Porqueddu C. (2016). Legumes for rainfed Mediterranean farming systems. Legume Perspect..

[5] Melis R.A.M., Franca A., Re G.A., Porqueddu C. (2018). Bio-agronomic characterization and implications on the potential use as forage of *Bituminaria bituminosa* and *B. morisiana* accessions. Grass Forage Sci..

[6] Smýkal P., Vernoud V., Blair M.W., Soukup A., Thompson R.D. (2014). The role of the testa during development and in establishment of dormancy of the legume seed. Front. Plant. Sci..

[7] Baskin J.M., Baskin C.C., Li X. (2000). Taxonomy, anatomy and evolution of physical dormancy in seeds. Plant Species Biol..

[8] Baskin J.M., Baskin C.C. (2004). A classification system for seed dormancy. Seed Sci. Res..

[9] Loi A., Cocks P.S., Howieson J.G., Carr S.J. (1999). Hardseededness and the pattern of softening in *Biserrula pelecinus* L., *Ornithopus compressus* L., and *Trifolium subterraneum* L. seeds. Aust. J. Agric. Res..

[10] Asci O.O., Acar Z., Ayan I., Basaran U., Mut H. (2011). Effect of pretreatments on seed germination rate of red clover (*Trifolium pratense* L.) populations. Afr. J. Agric. Res..

[11] Gresta F., Avola G., Onofri A., Anastasi U., Cristaudo A. (2011). When does hard coat impose dormancy in legume seeds? *Lotus* and *Scorpiurus* case study. Crop Sci..

[12] Gresta F., Avola G., Tuttobene R., Barrile V., Cristaudo A., Abbate V. (2011). The effect of fire on the dormancy break of three annual legume seeds. Ital. J. Agron..

[13] Rodrigues M.Â., Ferreira I.Q., Freitas S.L., Pires J.M., Arrobas M.P. (2015). Self-reseeding annual legumes for cover cropping in rainfed managed olive orchards. Span. J. Agric. Res..

[14] Renzi J.P., Duchoslav M., Brus J., Hradilová I., Pechanec V., Václavek T., Machalová J., Hron K., Verdier J., Smýkal P. (2020). Physical dormancy release in *Medicago truncatula* seeds is related to environmental variations. Plants.

[15] Gillikin J.W., Graham J.S. (1991). Purification and developmental analysis of the major anionic peroxidase from the seed coat of *Glycine max*. Plant Physiol..

[16] Hyde E.O.C., Mc Leavey A.M., Harris G.S. (1959). Seed development in ryegrass, and in red and white clover. N. Z. J. Agric. Res..

[17] Taylor G.B. (2005). Hardseededness in Mediterranean annual pasture legumes in Australia: A review. Aust. J. Agric. Res..

[18] Renzi J.P., Lasa J.C., Cantamutto M.A. (2011). Influence of maturity at harvest on the quality of alfalfa (*Medicago sativa* L.) seeds. Riarev. Investig. Agropecu..

[19] Baskin C.C., Baskin J.M. (2014). Seeds: Ecology, Biogeography, and Evolution of Dormancy and Germination.

[20] Rusdy M. (2017). A review on hardseedness and breaking dormancy in tropical forage legumes. Livest. Res. Rural Dev..

[21] Kimura E., Islam M.A. (2012). Seed Scarification Methods and their Use in Forage Legumes. Res. J. Seed Sci..

[22] Statwick J.M. (2016). Germination pretreatments to break hard-seed dormancy in *Astragalus cicer* L. (Fabaceae). PeerJ.

[23] Abbate V., Maugeri G., Cristaudo A., Gresta F. (2010). *Scorpiurus muricatus* L. subsp. *subvillosus* (L.) Thell., a potential forage legume species for a Mediterranean environment: A review. Grass Forage Sci..

[24] Guma I.R., Santos-Guerra A., Reyes-Betancort J.A., Padrón-Mederos M.A., Méndez P., González-Montelongo R. (2011). Perennial forage legumes endemic to the Canary Islands: Collection and ex situ conservation. Genet. Resour. Crop. Evol..

[25] Ventura M.R., Castanon J.I.R., Pieltain M.C., Flores M.P. (2004). Nutritive value of forage shrubs: *Bituminaria bituminosa*, *Rumex lunaria*, *Acacia salicina*, *Cassia sturtii* and *Adenocorpus foliosus*. Small Rumin. Res..

[26] Brullo S., Brullo C., Cambria S., Cristaudo A., Giusso del Galdo G. (2017). *Bituminaria antiatlantica* (Psoraleeae, Fabaceae), a new species from Morocco. PhytoKeys.

[27] Bogdanović S., Brullo C., Brullo S., Cambria S., Giusso del Galdo G. (2020). *Psoralea bituminosa* var. *Atropurpurea* (Psoraleeae, Fabaceae) from Morocco recognised as a distinct species in *Bituminaria*. Phytotaxa.

[28] Foster K., Ryan M.H., Real D., Ramankutty P., Lambers H. (2013). Seasonal and diurnal variation in the stomatal conductance and paraheliotropism of tedera (*Bituminaria bituminosa* var. *albomarginata*) in the field. Funct. Plant Biol..

[29] Foster K., Lambers H., Real D., Ramankutty P., Cawthray G., Ryan M. (2015). Drought resistance and recovery in mature *Bituminaria bituminosa* var. *Albomarginata*. Ann. Appl. Biol..

[30] Real D., Verbyla A.P. (2010). Maximizing genetic gains using a “plant” model in the Tedera (*Bituminaria bituminosa* var. *Albomarginata* and var. *crassiuscula*) breeding program in Australia. Options Méditerranéennessérie A.

[31] Poschenrieder C., Bech J., Llugany M., Pace A., Fenés E., Barceló J. (2001). Copper in plant species in a copper gradient in Catalonia (North East Spain) and their potential for phytoremediation. Plant Soil.

[32] del Río M., Font R., Almela C., Vélez D., Montoro R., De Haro Bailón A. (2002). Heavy metals and arsenic uptake by wild vegetation in the Guadiamar river area after the toxic spill of the Aznalcóllar mine. J. Biotechnol..

[33] Martínez S., Correal E., Real D., Ortuno A., del Río J.A., Awaad A.S., Govil J.N., Singh V.K. (2010). *Bituminaria bituminosa*: A source of furanocoumarins of pharmaceutical interest. Drug Plants. Recent Progress in Medicinal Plants.

[34] Pistelli L., Noccioli C., Appendino G., Bianchi F., Sterner O., Ballero M. (2003). Pterocarpans from *Bituminaria morisiana* and *Bituminaria bituminosa*. Phytochemistry.

[35] Martínez-Fernández D., Walker D.J., Romero-Espinar P., Flores P., del Río J.A. (2011). Physiological responses of *Bituminaria bituminosa* to heavy metals. J. Plant Phys..

[36] Llorent-Martínez E.J., Spínola V., Gouveia S., Castilhoa P.C. (2015). HPLC-ESI-MSn characterization of phenolic compounds, terpenoid saponins, and other minor compounds in *Bituminaria bituminosa*. Ind. Crop. Prod..

[37] Pecetti L., Mella M., Tava A. (2016). Variation in herbage biochemical composition among pitch trefoil (*Bituminaria bituminosa*) populations from Elba Island, Italy. J. Agric. Food Chem..

[38] Zennouhi O., El Mderssa M., Ibijbijen J., Bouiamrine E., Nassiri L. (2019). The use of *Bituminaria bituminosa* (L.) Stirton and microbial biotechnologies for restoration of degraded pastoral lands: The case of the Middle Atlas of Morocco. Int. J. Sci. Res. Environ. Sci..

[39] Correal E., Costa J., Hoyos A., Méndez P., Real D., Ríos S., Snowball R. (2008). Seed Production of *Bituminaria bituminosa*: Size, production, retention and germination capacity of the legumes. Options Méditerranéennessérie A.

[40] Beard C., Nichols P.G.H., Loo C., Michael P. (2014). Establishment of Tedera (Bituminaria Bituminosa var. Albomarginata).

[41] Barberá M., Flores M., Castanon J., Diazavila E., Ventura M. (2017). Germination characteristics of tedera (*Bituminaria bituminosa* var. *bituminosa*). Agrofor.

[42] Herranz J.M., Ferrandis P., Martínez-Sánchez J.J. (1998). Influence of heat on seed germination of seven Mediterranean Leguminosae species. Plant Ecol..

[43] Zennouhi O., Rfaki A., El Mderssa M., Bouiamrine E.H., Ibijbijen J., Nassiri L. (2018). Effect of salinity and temperature on the seed germination of *Bituminaria bituminosa* var. *bituminosa*. Int. J. Curr. Res..

[44] Reyes O., Trabaud L. (2009). Germination behaviour of 14 Mediterranean species in relation to fire factors: Smoke and heat. Plant Ecol..

[45] Minissale P., Brullo C., Brullo S., Giusso Del Galdo G., Sciandrello S. (2013). *Bituminaria basaltica* (Fabaceae), a new species from Italy. Phytotaxa.

[46] Gresta F., Cristaudo A., Onofri A., Restuccia A., Avola G. (2010). Germination response of four pasture species to temperature, light, and post-harvest period. Plant Biosyst..

[47] Gresta F., Cristaudo A., Trostle C., Anastasi U., Guarnaccia P., Catara S., Onofri A. (2018). Germination of guar (*Cyamopsis tetragonoloba* (L.) Taub.) genotypes with reduced temperature requirements. Aust. J. Crop Sci..

[48] Souza De F.H.D., Marcos-Filho J. (2001). The seed coat as a modulator of seed-environment relationships in Fabaceae. Braz. J. Bot..

[49] Ellis R.H., Hong T.D., Roberts E.H. (1987). The development of desiccation tolerance and maximum seed quality during seed maturation in six Grain legumes. Ann. Bot..

[50] Mai-Hong T., Hong T.D., Hien N.T., Ellis R.H. (2003). Onset of germinability, desiccation tolerance and hardseededness in developing seeds of *Peltophorum pterocarpum* (DC) K. Heyne (Caesalpinioideae). Seed Sci. Res..

[51] Duguma B., Kang B.T., Okali D.U.U. (1988). Factors affecting germination of *Leucaena leucocephala*. Seed Sci. Res..

[52] Samarah N.H., Allataifeh N., Turk M., Tawaha A.R. (2003). Effect of maturity stage on germination and dormancy of fresh and air-dried seeds of bitter vetch (*Vicia ervilia* L.). N. Z. J. Agric. Res..

[53] Cristaudo A., Gresta F., Avola G., Miano V. (2008). Germination capability of immature seeds of *Lotus ornithopodioides* L. and *Scorpiurus subvillosus* L.. Options Méditerranéennessérie A.

[54] Argel P.J., Paton C.J., Loch D.S., Ferguson J.E. (1999). Overcoming legume hardseededness. Forage Seed Production.

[55] Venier P., Funes G., Carrizo García C. (2012). Physical dormancy and histological features of seeds of five *Acacia* species (Fabaceae) from xerophytic forests in central Argentina. Flora.

[56] Galussi A.A., Moya M.E., Jimenez-Lopez J.C. (2017). Anatomical and chemical insights into the white clover (*Trifolium repens* L.) seed coat associated to water permeability. Advances in Seed Biology.

[57] Hradilová I., Duchoslav M., Brus J., Pechanec V., Hýbl M., Kopecký P., Smržová L., Štefelová N., Vaclávek T., Bariotakis M. (2019). Variation in wild pea (*Pisum sativum* subsp. *elatius*) seed dormancy and its relationship to the environment and seed coat traits. PeerJ.

[58] Russi L., Cocks P.S., Roberts E.H. (1992). Coat thickness and hard-seededness in some *Medicago* and *Trifolium* species. Seed Sci. Res..

[59] Marbach I., Mayer A.M. (1974). Permeability of seed coats to water as related to drying conditions and metabolism phenolics. Plant Physiol..

[60] Werker E., Marbach I., Mayer A.M. (1979). Relation between the anatomy of the testa, water permeability and the presence of phenolics in the genus Pisum. Ann. Bot..

[61] Galussi A.A., Argüello J.A., Cerana M.M., Maximino M., Moya M.E. (2016). Anatomical and chemical characteristics of the seed coat of *Medicago sativa* L. (alfalfa) cv. Baralfa 85 seeds and their association with seed dormancy. Phyton Int. J. Exp. Bot..

[62] Hu X.W., Wang Y.R., Wu Y.P., Baskin C.C. (2009). Role of the lens in controlling water uptake in seeds of two Fabaceae (Papilonoideae) species treated with sulphuric acid and hot water. Seed Sci. Res..

[63] Gama-Arachchige N.S., Baskin J.M., Geneve R.L., Baskin C.C. (2013). Identification and characterization of ten new water-gaps in seeds and fruits with physical dormancy and classification of water-gap complexes. Ann. Bot..

[64] Yaklich R.W., Vigil E.L., Wergin W. (1986). Pore development and seed coat permeability in soybean. Crop Sci..

[65] Ragus L.N. (1987). Role of water absorbing capacity in soybean germination and seedling vigour. Seed Sci. Technol..

[66] Souza De F.H.D., Marcos-Filho J., Nogueira M.C.S. (1996). Características físicas das sementes de *Calopogonium mucunoides* associadas a absorção de água e qualidade fisiológica. I. Tamanho. Rev. Bras. Sementes.

[67] Rodrigues-Junior A.G., Mello A.C.M.P., Baskin C.C., Baskin J.M., Oliveira D.M.T., Garcia Q.S. (2018). Why large seeds with physical dormancy become nondormant earlier than small ones. PLoS ONE.

[68] Efron B. (1981). Nonparametric estimates of standard error: The Jackknife, the bootstrap and other methods. Biometrika.

[69] R Core Team (2020). R: A Language and Environment for Statistical Computing.

