# Supplementary material for: Seed Dormancy Breaking and Germination in Bituminaria basaltica and B. bituminosa (Fabaceae)
Source: Plants (Basel). 2020 Aug 27;9(9):1110. doi: 10.3390/plants9091110 (PMC7570033; doi:10.3390/plants9091110)
Supplement: Supplementary file 1 [file plants-09-01110-s001.pdf]

plants

Supplementary files

Table S1. Final germination percentage (FGP) with standard error (*SE*) at different incubation temperatures (15, 20, 25, 20/15, 25/20 °C) in *B. basaltica* and *B. bituminosa*, after different immersion times in H_2SO_4. Percentages were derived by back-transformation of model parameters, following a GLM fit (see materials and methods for details).

**Immersion Time (min)**	**Temperature (°C)**	***Bituminaria basaltica***		***Bituminaria bituminosa***	
		**FPG**	***SE***	**FPG**	***SE***
10	15	5.0	*4.0*	22.5	*7.6*
20	15	17.5	*6.9*	40.0	*8.9*
30	15	35.0	*8.7*	57.5	*9.0*
40	15	65.0	*8.7*	85.0	*6.5*
50	15	75.0	*7.9*	77.5	*7.6*
10	20	7.5	*4.8*	17.5	*6.9*
20	20	22.5	*7.6*	62.5	*8.8*
30	20	37.5	*8.8*	65.0	*8.7*
40	20	75.0	*7.9*	77.5	*7.6*
50	20	62.5	*8.8*	87.5	*6.0*
10	20/15	10.0	*5.5*	25.0	*7.9*
20	20/15	25.0	*7.9*	42.5	*9.0*
30	20/15	32.5	*8.5*	62.5	*8.8*
40	20/15	82.5	*6.9*	87.5	*6.0*
50	20/15	77.5	*7.6*	95.0	*4.0*
10	25	7.5	*4.8*	17.5	*6.9*
20	25	20.0	*7.3*	40.0	*8.9*
30	25	45.0	*9.1*	67.5	*8.5*
40	25	65.0	*8.7*	65.0	*8.7*
50	25	80.0	*7.3*	92.5	*4.8*
10	25/20	10.0	*5.5*	25.0	*7.9*
20	25/20	22.5	*7.6*	35.0	*8.7*
30	25/20	30.0	*8.3*	55.0	*9.1*
40	25/20	72.5	*8.1*	65.0	*8.7*
50	25/20	95.0	*4.0*	67.5	*8.5*
Significance of effects (p - value)					
Immersion time		<2e-16		<2e-16	
Temperature		0.5886		0.08134	
Immersion time × Temperature		0.5744		0.31317	

Table S2. Final germination percentage (FGP) with standard error (*SE*) at different incubation temperatures (10, 15, 20, 25, 20/15, 25/20 °C) in *B. basaltica* and *B. bituminosa*, after different immersion times in hot constant water (100 and 70 °C). Percentages were derived by back-transformation of model parameters, following a GLM fit (see materials and methods for details).

Immersion Time (min)	Temperature (°C)	Treatment	*Bituminaria basaltica*		*Bituminaria bituminosa*	
			**FPG**	***SE***	**FPG**	***SE***
2	10	70 °C	20.0	*4.6*	48.0	*7.4*
4	10	70 °C	28.0	*5.1*	56.0	*7.4*
6	10	70 °C	26.0	*5.0*	70.0	*6.8*
8	10	70 °C	30.0	*5.2*	78.0	*6.1*
10	10	70 °C	22.0	*4.7*	80.0	*5.9*
2	15	70 °C	28.0	*5.1*	40.0	*7.3*
4	15	70 °C	26.0	*5.0*	64.0	*7.1*
6	15	70 °C	16.0	*4.2*	70.0	*6.8*
8	15	70 °C	26.0	*5.0*	76.0	*6.3*
10	15	70 °C	22.0	*4.7*	88.0	*4.8*
2	20	70 °C	32.0	*5.3*	48.0	*7.4*
4	20	70 °C	22.0	*4.7*	70.0	*6.8*
6	20	70 °C	40.0	*5.6*	76.0	*6.3*
8	20	70 °C	40.0	*5.6*	86.0	*5.1*
10	20	70 °C	30.0	*5.2*	88.0	*4.8*
2	20/15	70 °C	30.0	*5.2*	58.0	*7.3*
4	20/15	70 °C	28.0	*5.1*	66.0	*7.0*
6	20/15	70 °C	28.0	*5.1*	82.0	*5.7*
8	20/15	70 °C	34.0	*5.4*	82.0	*5.7*
10	20/15	70 °C	36.0	*5.5*	86.0	*5.1*
2	25	70 °C	36.0	*5.5*	54.0	*7.4*
4	25	70 °C	30.0	*5.2*	62.0	*7.2*
6	25	70 °C	50.0	*5.7*	88.0	*4.8*
8	25	70 °C	30.0	*5.2*	84.0	*5.4*
10	25	70 °C	40.0	*5.6*	94.0	*3.5*
2	25/20	70 °C	32.0	*5.3*	62.0	*7.2*
4	25/20	70 °C	26.0	*5.0*	66.0	*7.0*
6	25/20	70 °C	16.0	*4.2*	84.0	*5.4*
8	25/20	70 °C	28.0	*5.1*	88.0	*4.8*
10	25/20	70 °C	30.0	*5.2*	84.0	*5.4*
2	10	100 °C	4.0	*2.2*	66.0	*7.0*
4	10	100 °C	0.0	*0.0*	44.0	*7.4*
6	10	100 °C	2.0	*1.6*	44.0	*7.4*
8	10	100 °C	0.0	*0.0*	10.0	*4.4*
10	10	100 °C	0.0	*0.0*	10.0	*4.4*
2	15	100 °C	8.0	*3.1*	64.0	*7.1*
4	15	100 °C	4.0	*2.2*	50.0	*7.4*
6	15	100 °C	2.0	*1.6*	34.0	*7.0*
8	15	100 °C	0.0	*0.0*	26.0	*6.5*
10	15	100 °C	0.0	*0.0*	14.0	*5.1*
2	20	100 °C	6.0	*2.7*	64.0	*7.1*
4	20	100 °C	2.0	*1.6*	38.0	*7.2*
6	20	100 °C	4.0	*2.2*	26.0	*6.5*

8	20	100 °C	0.0	*0.0*	24.0	*6.3*
10	20	100 °C	2.0	*1.6*	28.0	*6.7*
2	20/15	100 °C	12.0	*3.7*	64.0	*7.1*
4	20/15	100 °C	2.0	*1.6*	38.0	*7.2*
6	20/15	100 °C	0.0	*0.0*	28.0	*6.7*
8	20/15	100 °C	0.0	*0.0*	12.0	*4.8*
10	20/15	100 °C	0.0	*0.0*	20.0	*5.9*
2	25	100 °C	4.0	*2.2*	56.0	*7.4*
4	25	100 °C	0.0	*0.0*	48.0	*7.4*
6	25	100 °C	2.0	*1.6*	22.0	*6.1*
8	25	100 °C	0.0	*0.0*	32.0	*6.9*
10	25	100 °C	0.0	*0.0*	16.0	*5.4*
2	25/20	100 °C	6.0	*2.7*	74.0	*6.5*
4	25/20	100 °C	0.0	*0.0*	44.0	*7.4*
6	25/20	100 °C	0.0	*0.0*	32.0	*6.9*
8	25/20	100 °C	2.0	*1.6*	14.0	*5.1*
10	25/20	100 °C	0.0	*0.0*	8.0	*4.0*

Significance of effects (p - value)		
Immersion time	0.12684	0.08206
Temperature	0.00143	0.47115
Treatment	0.00001	0.00001
Immersion time × Temperature	0.07757	0.37989
Immersion time × Treatment	0.00001	0.00001
Temperature × Treatment	0.16406	0.10386
Immersion time × Temperature × Treatment	0.31764	0.49370

Table S3. Final germination percentage (FGP) with standard error (*SE*) in *B. basaltica and B. bituminosa* at 25/20 °C incubation temperature, after different immersion times in hot constant water (70, 80, 90 and 100 °C). Percentages were derived by back-transformation of model parameters, following a GLM fit (see materials and methods for details).

**Immersion Time (min)**	**Treatment**	***Bituminaria basaltica***		***Bituminaria bituminosa***	
		**FPG**	***SE***	**FPG**	***SE***
2	70 °C	30.0	*3.2*	62.0	*4.6*
4	70 °C	26.0	*3.0*	70.0	*4.4*
6	70 °C	24.0	*2.9*	76.0	*4.1*
8	70 °C	26.0	*3.0*	86.0	*3.3*
10	70 °C	30.0	*3.2*	84.0	*3.5*
2	80 °C	50.0	*3.4*	68.0	*4.5*
4	80 °C	50.0	*3.4*	68.0	*4.5*
6	80 °C	44.0	*3.4*	70.0	*4.4*
8	80 °C	30.0	*3.2*	74.0	*4.2*
10	80 °C	50.0	*3.4*	80.0	*3.8*
2	90 °C	22.0	*2.8*	70.0	*4.4*
4	90 °C	14.0	*2.4*	66.0	*4.5*
6	90 °C	6.0	*1.6*	56.0	*4.7*
8	90 °C	8.0	*1.9*	58.0	*4.7*
10	90 °C	8.0	*1.9*	46.0	*4.8*
2	100 °C	6.0	*1.6*	74.0	*4.2*
4	100 °C	0.0	*0.0*	44.0	*4.7*
6	100 °C	0.0	*0.0*	32.0	*4.5*
8	100 °C	2.0	*1.0*	14.0	*3.3*
10	100 °C	0.0	*0.0*	8.0	*2.6*
Significance of effects (p - value)					
Immersion time		0.0001448		0.002756	
Treatment		4.21E-16		2.37E-11	
Immersion time × Treatment		0.0008424		4.08E-07	

Table S4. Final germination percentage (FGP) with standard error (*SE*) in *B. basaltica and B. bituminosa* at 20/15 and 25/20 °C incubation temperatures, after different immersion times in boiling water gradually cooling up to room temperature. Percentages were derived by back-transformation of model parameters, following a GLM fit (see materials and methods for details).

**Immersion Time (min)**	**Temperature (°C)**	***Bituminaria basaltica***		***Bituminaria bituminosa***	
		**FPG**	***SE***	**FPG**	***SE***
5	20/15	20.0	*4.9*	60.0	*6.7*
10	20/15	30.0	*5.6*	55.0	*6.8*
15	20/15	40.0	*5.9*	57.5	*6.8*
30	20/15	25.0	*5.3*	62.5	*6.6*
1440	20/15	27.5	*5.4*	60.0	*6.7*
5	25/20	50.0	*6.1*	67.5	*6.4*
10	25/20	30.0	*5.6*	65.0	*6.5*
15	25/20	40.0	*5.9*	52.5	*6.9*
30	25/20	42.5	*6.0*	77.5	*5.7*
1440	25/20	32.5	*5.7*	60.0	*6.7*
Significance of effects (p - value)					
Immersion time		0.39669		0.2533	
Temperature		0.00673		0.1994	
Immersion time ×Temperature		0.05522		0.5383	

Table S5. Final germination percentage (FGP) with standard error (*SE*) in *B. basaltica and B. bituminosa* at different incubation temperatures (10, 15, 20, 25, 15/10, 20/10, 20/15, 25/20 °C); pods untreated and after mechanical scarification. Percentages were derived by back-transformation of model parameters, following a GLM fit (see materials and methods for details).

**Treatment**	**Temperature (°C)**	***Bituminaria basaltica***		***Bituminaria bituminosa***	
		**FPG**	***SE***	**FPG**	***SE***
mechanical	10	100.0	*0.0*	100.0	*0.0*
mechanical	15	100.0	*0.0*	100.0	*0.0*
mechanical	20	100.0	*0.0*	100.0	*0.0*
mechanical	25	100.0	*0.0*	100.0	*0.0*
mechanical	15/10	100.0	*0.0*	99.0	*0.7*
mechanical	20/10	99.0	*0.7*	99.0	*0.7*
mechanical	20/15	99.5	*0.5*	98.5	*0.9*
mechanical	25/20	98.0	*1.0*	99.0	*0.7*
untreated	10	9.3	*2.5*	14.4	*2.3*
untreated	15	8.0	*2.3*	15.6	*2.4*
untreated	20	10.7	*2.6*	14.4	*2.3*
untreated	25	5.3	*1.9*	14.0	*2.3*
untreated	15/10	5.5	*1.7*	11.5	*1.7*
untreated	20/10	6.5	*1.8*	7.0	*1.3*
untreated	20/15	7.2	*1.7*	8.0	*1.3*
untreated	25/20	11.6	*2.1*	9.6	*1.4*
		Significance of effects (p - value)			
Treatment		2.00E-16		2.20E-16	
Temperature		0.46231		0.001042	
Treatment × Temperature		0.07654		0.725584	
